# Osteoblast derived extracellular vesicles induced by dexamethasone: A novel biomimetic tool for enhancing osteogenesis *in vitro*

**DOI:** 10.3389/fbioe.2023.1160703

**Published:** 2023-03-10

**Authors:** Xing Zhang, Qun Zhao, Nan Zhou, Yu Liu, Kang Qin, Eva Miriam Buhl, Xinhong Wang, Frank Hildebrand, Elizabeth R. Balmayor, Johannes Greven

**Affiliations:** ^1^ Department of Orthopedics, Trauma and Reconstructive Surgery, University Hospital RWTH Aachen, Aachen, Germany; ^2^ Electron Microscopy Facility, Institute of Pathology and Medical Clinic II, University Hospital RWTH Aachen, Aachen, Germany; ^3^ Department of Orthopedics, The Affliated Huai’an Hospital of Xuzhou Medical University, Huai’an Second People’s Hospital, Huai’an, Jiangsu, China

**Keywords:** extracellular vesicles, dexamethasone stimulation, intercellular crosstalk, osteoblastic differentiation, osteogenesis, bone regeneration

## Abstract

Extracellular vesicles (EVs) are newly appreciated communicators involved in intercellular crosstalk, and have emerged as a promising biomimetic tool for bone tissue regeneration, overcoming many of the limitations associated with cell-based therapies. However, the significance of osteoblast-derived extracellular vesicles on osteogenesis has not been fully established. In this present study, we aim to investigate the therapeutic potential of extracellular vesicles secreted from consecutive 14 days of dexamethasone-stimulated osteoblasts (OB-EV_Dex_) to act as a biomimetic tool for regulating osteogenesis, and to elucidate the underlying mechanisms. OB-EV_dex_ treated groups are compared to the clinically used osteo-inductor of BMP-2 as control. Our findings revealed that OB-EV_Dex_ have a typical bilayer membrane nanostructure of, with an average diameter of 178 ± 21 nm, and that fluorescently labeled OB-EV_Dex_ were engulfed by osteoblasts in a time-dependent manner. The proliferation, attachment, and viability capacities of OB-EV_Dex_-treated osteoblasts were significantly improved when compared to untreated cells, with the highest proliferative rate observed in the OB-EV_Dex_ + BMP-2 group. Notably, combinations of OB-EV_Dex_ and BMP-2 markedly promoted osteogenic differentiation by positively upregulating osteogenesis-related gene expression levels of *RUNX2*, *BGLAP*, *SPP1*, *SPARC*, *Col 1A1*, and *ALPL* relative to BMP-2 or OB-EV_Dex_ treatment alone. Mineralization assays also showed greater pro-osteogenic potency after combined applications of OB-EV_Dex_ and BMP-2, as evidenced by a notable increase in mineralized nodules (calcium deposition) revealed by Alkaline Phosphatase (ALP), Alizarin Red Alizarin Red staining (ARS), and von Kossa staining. Therefore, our findings shed light on the potential of OB-EV_Dex_ as a new therapeutic option for enhancing osteogenesis.

## 1 Introduction

Bone defects, caused by traumatic injuries, age-associated disorders, infections, and surgical resections, have been a global medical and socioeconomic challenge that severely impairs the natural bone healing process (Dimitriou et al., 2011; El-Rashidy et al., 2017). The current “gold standard” treatment algorithm in clinical settings for augmenting bone regeneration involves applications of autologous and allogeneic bone grafting (Roseti et al., 2017). Mostly these approaches have positive clinical outcomes; however, they are associated with several limitations including limited availability, risk of disease transmission, donor site morbidity, and unexpected immunoreaction, among others that may result in malunion (Ng et al., 2017; Schmidt, 2021). Bone morphogenetic protein-2 (BMP-2), a potent osteoinductive cytokine, was believed to be “near-perfect” in achieving bone induction; however, it simultaneously has accrued a worrisome side effect profile, such as ectopic bone formation, osteoclast-mediated bone resorption, and inappropriate adipogenesis (James et al., 2016). Thus, there is an urgent need to develop alternative therapeutic strategies that can mitigate risks and simultaneously augment bone regeneration.

Cell-based therapies are attractive as a promising alternative solution since they attempt to mimic and improve the body’s natural regenerative potential for bone repair (Buzhor et al., 2014). Stem cell-based therapies have been broadly reported, however, clinical success is restricted owing to insurmountable hurdles associated with high costs, ethical concerns, low homing efficacy of transplanted cells, and variations in differentiation capacities (Walmsley et al., 2016; Ho-Shui-Ling et al., 2018). Faced with these limitations, the development of novel biological approaches for bone regeneration that retain many of the advantages of cell-based approaches, with the aim of achieving functional osteogenesis, is of vital importance.

Extracellular vesicles (EVs), one of the cell-secreted factors, have sparked increasing interest as a potential therapeutic tool for regenerative medicine in the past decades (van Niel et al., 2018; Liu et al., 2022). EVs are a group of cell-derived vesicles with sizes ranging from 30 to 1,000 nm, that can be broadly categorized into three major subtypes based on the putative biological pathways: Exosomes, microvesicles, and apoptotic bodies (Li et al., 2021). Nowadays, the biological implications of EVs dramatically evolved, switching from the original concept of secreting cellular wastes/debris to a regulated means of biological information exchange based on cellular needs and status. Accumulating evidence indicated that EVs play a crucial role in intercellular communication by transporting complex cargoes, such as nucleic acids (mRNA or microRNA), functional proteins, lipids, and biologically active molecules to target cells (Théry et al., 2009; Lin et al., 2022; van Niel et al., 2022). Thereby regulating tissue metabolism, homeostasis, and development by so-called horizontal transfer driven by receptor-ligand interactions and endocytosis (Colombo et al., 2014). Such unique features make EVs a potential candidate for restoring bone defects and augmenting bone regeneration.

In recent years, EVs have exhibited great promise in regenerative medicine as biomimetic tools for inducing lineage-specific stem cell differentiation (Alqurashi et al., 2021; Hade et al., 2021). Besides, the considerable utility of stem cells-derived EVs in improving osteogenesis has been extensively reported (Narayanan et al., 2016; Huang et al., 2017; Pizzicannella et al., 2019). It has been reported that EVs exerted a crucial role in amplifying microRNA (miR-29a, miR-3, etc.) cargoes transport to recipient cells, thereby enhancing their osteogenic differentiation (Huang et al., 2017; Hu et al., 2020; Lu et al., 2020). A recent study revealed that osteoblast-derived EVs play a physiologic role in interacting with other cells (monocytes and osteoclasts) in the bone microenvironment (Cappariello et al., 2018). Moreover, Davies et al. (2017) reported the favorable biological effect of EVs derived from osteoblasts, a potent osteo-inductive capacity that elicits enhanced stem cell mineralization to modulate osteogenesis. These findings suggest an effective intercellular communication mediated by EVs in the osteogenic microenvironment.

Numerous studies have evaluated the specific roles of MSC-derived EVs; however, it is surprising that, to date, only a very limited number of studies investigate the role of osteoblast-derived EVs. Previous literature reported that dexamethasone can dramatically enhance the middle-to-late stage of osteoblastic differentiation of hBMSC (Martins et al., 2010); EVs secreted from mineralizing osteoblasts (late differentiation) have also been identified to have a high pro-osteogenic potency (Cui et al., 2016). In this context, we put forward the hypothesis that EVs derived from synchronized late-stage osteoblast differentiation caused by 14 days of dexamethasone stimulation (OB-EV_Dex_) would present an exceptional osteo-promoting capacity for improved osteogenesis.

In this study, we aim to investigate the osteogenic potential of dexamethasone-induced OB-EV_Dex_ on osteogenesis and explore their potential utility as biomimetic tools to accelerate bone augmentation. Furthermore, the therapeutic role of OB-EV_Dex_ was validated by examining the proliferation, viability, growth, and osteoblastic differentiation capacities of osteoblasts after OB-EV_Dex_ treatment. This study may open a new horizon for exploring EVs in regenerative medicine for restoring bone defects in a shorter period of treatment time or bringing up the possibility of non-union treatment.

## 2 Materials and methods

### 2.1 Cell culture under normal conditions and osteogenic conditions

Porcine osteoblasts (OB) were isolated according to a previously published protocol with a slight modification (Bakker and Klein-Nulend, 2012; Perpétuo et al., 2019). Osteoblast cells were routinely cultured in growth medium (GM) containing basal Dulbecco’s Modified Eagle Medium (DMEM, Invitrogen, Carlsbad, CA, United States) supplemented with 10% fetal bovine serum (FBS), 1% double-antibiotics (penicillin/streptomycin), and maintained at 37°C in a humidified 5% CO_2_ atmosphere. Isolated cells were passaged until passage 3–5 for subsequent experiments. For osteogenic induction, osteoblasts were maintained and treated with a common osteogenic induction medium (OM) supplemented with 100 nM dexamethasone, 10 mM *β*-glycerophosphate, and 100 μM L-ascorbic acid-2-phosphate.

Osteoblasts were seeded on the commercially available particulate bone graft substitutes (biphasic calcium phosphate, BCP) with the particle size of 0.5–2 mm (maxresorb^Ⓡ^, botiss biomaterials GmbH, Zossen, Germany). BCP granules have a chemical composition of 60% hydroxyapatite (HA) and 40% *β*-tricalcium phosphate (β-TCP) and displayed good stability with a porosity of about 80%, interconnectivity, and rough surfaces, which is beneficial to the adhesion and growth of seeded osteoblasts. For sterilization, the BCP granules were rinsed with sterile deionized water, and then sterilized by overnight incubation in 70% ethanol as well as 1 h of UV exposure.

### 2.2 Isolation and characterization of OB-EV_Dex_

#### 2.2.1 Isolation

Prior to OB-EV_Dex_ extraction, porcine osteoblasts were plated at an initial density of 40,000 cells/well in the 6-well plates. Afterwards, dexamethasone (500 nM) was supplemented into the GM medium to sustainably stimulate osteoblast cells for 14 consecutive days in order to generate OB-EV_Dex_. Only dexamethasone as an osteogenic stimulant was chosen. Commonly employed *β*-glycerophosphate and L-ascorbic acid-2-phosphate were not used in our study. Thereby, the obtained results will be correlated specifically to dexamethasone later on. At day 14, dexamethasone-stimulated osteoblast cells were rinsed with a serum-free medium and maintained under serum-free conditions for another 24 h. Subsequently, the dexamethasone-stimulated conditioned medium was collected and subjected to a series of differential centrifugation at 300 g, ×2,000 g, and ×5,000 g for 15 min at 4°C, respectively, to eliminate remnant cells, followed by filtration through a 0.22 μm filter for removal of excess cell debris. The supernatant was harvested and centrifuged twice at ×20,000 g in a sterile Ultra-Clear™ tube (Beckman Coulter, Brea, CA, United States) for 90 min for purification. Finally, OB-EV_Dex_ were pelleted by ultracentrifugation at ×100,000 g for 90 min, resuspended in filtered PBS, and stored at −80°C for further use.

#### 2.2.2 Characterization examinations of OB-EV_Dex_

The concentration and size distribution of OB-EV_Dex_ were determined by transmission electron microscopy (TEM) and nanoparticle tracking analysis (NTA) with a NanoSight NS300 (Malvern, Worcestershire, United Kingdom) with 545 nm laser. For NTA, the OB-EV_Dex_ suspension was assessed using a NanoSight NS-300 instrument to acquire size distribution plots and corresponding concentration (particles/mL). For TEM observations, the harvested OB-EV_Dex_ resuspended in 10 μL of Hepes buffer (0.1 M, pH 7.4) were allowed to absorb onto the glow discharged formvar-carbon-coated nickel grids (Maxtaform, 200 mesh, Plano, Wetzlar, Germany) for 10 min. After that, samples on grids were stained by placing shortly on a drop of 0.5% uranyl acetate in distilled water (DW). After air drying, samples were examined using a TEM LEO 906 (Carl Zeiss, Oberkochen, Germany), operating at an acceleration voltage of 60 kV. To maintain consistency, the isolated OB-EV_Dex_ were resuspended in 100 μL of DPBS after which an equal number and concentration of OB-EV_Dex_ were ensured for each experiment.

### 2.3 Cytotoxicity assay of bone graft granules and OB-EV_Dex_

The Cell Counting Kit-8 (CCK-8, Sigma-Aldrich, Steinheim, Germany) assay was performed to assess the potential cytotoxicity of bone graft BCP granules towards OB cells in the presence or absence of OB-EV_Dex_. The concentration of EVs used was around 5–6 × 10^9^ particles/mL. Osteoblast cells were seeded in 96-well plates with a density of 5,000 cells/well and incubated at 37°C overnight to adhere to plates, after which they were exposed to varying concentrations of DMEM medium suspension containing BCP granules (25, 50, 75, 100, 200, 500, 1,000, 2000, and 3,000 μg/mL) and cultured for 1, 4, and 7 days. Afterward, DMEM medium was removed, and 10 μL of CCK-8 solutions was introduced into each well and incubated for another 2 h. Parallel sets of wells with freshly cultured, non-treated cells served as negative controls. Optical densities were determined at 450 nm wavelength through a microplate reader (Infinite M200, Tecan, Switzerland). Besides, the absorbance of cells simultaneously treated with BCP granules and OB-EV_Dex_ was measured following the same testing procedures.

### 2.4 OB-EV_Dex_ cellular internalization

With regards to endocytosis experiments, isolated OB-EV_Dex_ were marked with a molecular fluorescent lectin probe, Wheat Germ Agglutinin Conjugates (WGA, Alexa Fluor^®^ 488 conjugate, Invitrogen, Carlsbad, CA, United States), which selectively binds to the sialic acid on the membranes for the labeling. Prior to the experiments, osteoblasts at a density of 5 × 10^4^/dish were seeded onto each confocal dish (VWR GmbH, Darmstadt, Germany) and incubated for 24 h. In brief, OB-EV_Dex_ were centrifuged and collected, after which recommended amounts of probe solution were applied for labeling EVs. Labeling of the EVs was performed following the instructions provided with the lectin probe. Fluorescently labeled OB-EV_Dex_ were rinsed thrice with DPBS and centrifuged at ×20,000 g for 90 min to remove excess probes. Fluorescently labeled OB-EV_Dex_ were dispersed in DMEM and then supplemented into cell-seeded confocal dishes at 37°C for 1, 4, 12, and 72 h incubation. Finally, the amount of fluorescently labeled OB-EV_Dex_ internalized into cells was evaluated *via* a confocal laser scanning microscope (CLSM, LSM 710, Carl Zeiss MicroImaging GmbH, Jena, Germany).

### 2.5 Cell attachment and proliferation under various treatment groups

Sterilized BCP granules were placed on confocal dishes after which OB cells were seeded at a density of 20,000 cells/well. Cells randomly received the following treatments: 1) only BCP granules (control), 2) BMP-2 loaded BCP granules (BMP-2), 3) BCP granules plus OB-EV_Dex_ (OB-EV_Dex_), and 4) BMP-2 loaded BCP granules plus OB-EV_Dex_ (BMP-2+OB-EV_Dex_). After various treatments for 1, 4, and 7 days, cell attachment and proliferation of OB cells were evaluated. Firstly, calcein AM/DAPI staining was performed. In brief, cells were stained using 5 μM of fluorescence dye (Calcein AM, PromoCell GmbH, Heidelberg, Germany) for 40 min without light. DAPI staining dye (Invitrogen, Karlsruhe, Germany) was used at 10 μg/mL final concentration. Cells were stained at the indicated time-points and subsequently visualized *via* a fiuorescence microscope (Leica DM IRB, Wetzlar, Germany). In addition, a CCK-8 assay was also performed. OB cells were cultured on BCP granules in 48-well plates at an initial density of 2 × 10^4^ cells/well and subjected to four above-mentioned treatments for 1, 4, and 7 days prior to evaluation with the CCK-8 kit.

### 2.6 Cell viability analysis

OB cells were cultured on BCP granules under the four above-mentioned treatments (please see 2.5) at an initial density of 2 × 10^4^ cells/well for 1, 4, and 7 days. At each predetermined time point, the medium was removed, the cells were rinsed, and labeled with the Live/Dead Cell Viability Assay kit (PromoCell GmbH, Heidelberg, Germany) containing 2 μM calcein AM and 4 μM ethidium homodimer-III (EthD-III), as per the manufacturer’s instructions. Images were captured by a fiuorescent microscope fitted with appropriate exciter and emitter filters to evaluate live and dead cells, which had been labeled with green and red fluorescence, respectively. Cell survival rate was calculated according to the following equation: Cell survival rate (%) = [N_live_/N_dead_ + N_live_] × 100%, where N_live_ is the number of live cells, and N_dead_ is the number of dead cells.

### 2.7 Evaluation of cell growth and morphology

After incubation for 7 days, we assessed cell morphologies on BCP granules under the four applied treatments. For this evaluation, cells were seeded at an initial density of 2 × 10^4^ cells/well. Cell morphology was investigated through cytoskeleton staining and scanning electron microscopy (SEM, ESEM XL 30 FEG, FEI, Eindhoven, Netherlands). With regards to cytoskeleton staining, OB cells were subjected to co-staining with Rhodamine Phalloidin (ThermoFisher, Grand Island, NY, United States) and DAPI, which aid in visualizing F-actin and cell nuclei. Briefly, cells were rinsed with DPBS, fixed in 4% paraformaldehyde, followed by permeabilizing with 0.1% Triton X-100 and blocking with 1% BSA solution. After that, cells were co-stained with the indicated fluorescent regents and visualized *via* a CLSM under Ex/Em (540/565 nm) wavelength. For SEM observation, OB cells in various treatment groups were treated with an SEM fixation solution without light and dehydrated through varying gradient ethanol. Lastly, the morphologies of OB cells were detected *via* SEM after spray-coating with 4 nm thick gold.

### 2.8 Osteogenic differentiation and osteogenic potential validation

#### 2.8.1 Alkaline Phosphatase (ALP) activity assay and ALP staining

OB cells were cultured on the BCP granules in OM medium and subjected to four different treatments for indicated durations. OM medium was chosen to induce osteogenic differentiation. ALP activities of OB cells in the various studied groups were quantitatively determined using an ALP Activity Assay Kit (PromoKine, PromoCell Gmbh, Heidelberg, Germany), which uses pnitrophenyl phosphate (pNPP) as the phosphatase substrate. Besides, the microplate reader was used for measuring the absorbance (O.D. value) at 405 nm. The ALP activities were calculated as the following equation: ALP activity = A/V/T, whereby A is the amount of pNP generated by OB cells, V is the volume of cell samples, and T is the reaction time. ALP staining was performed at 7 and 14 days after culture. Initially, OB cells under the various treatments were rinsed thrice with DPBS and fixed for 2 min at room temperature, followed by addition of ALP staining solution (Abcam, Cambridge, United Kingdom) in darkness for 30 min.

#### 2.8.2 *In vitro* mineralization assessment

After incubation for 14 days, Alizarin red staining (ARS, Thermo Fisher, Waltham, MA, United States) and von Kossa staining (Sigma-Aldrich, St. Louis, MO, United States) were performed to evaluate mature mineralized nodules during late phase of osteogenic differentiation (Kwon et al., 2014). For ARS staining, OB cells subjected to the various studied treatments were washed using DPBS, fixed in 2% paraformaldehyde, and then stained with ARS working solution. Culture plates were imaged *via* an optical microscope. Mineralized nodules are shown as a dark red center and light red peripheral area. For von Kossa staining, OB cells were rinsed with DPBS and fixed in 2% paraformaldehyde for 15 min, followed by staining with a freshly prepared 5% silver nitrate solution for 40 min under UV exposure. After that, cells were immersed in fresh 5% sodium carbonate to indicate minerals and matrix of calcium deposits. Lastly, positive calcium deposits with brownish-blackish color were photographed using an optical microscope.

### 2.9 Expressions of related osteogenic marker genes during osteogenic differentiation

The OB cells under four different groups at an initial density of 20,000 cells/well were maintained in the osteogenic induction medium in confocal culture dishes for 0, 3, 7, and 14 days. At various predetermined time points, cells were obtained, and their osteogenic differentiation was assessed by examining the expression levels of osteogenesis-related genes of type 1A1 collagen (*Col 1A1*), *ALPL*, osteocalcin (*BGLAP*), osteopontin (*SPP1)*, osteonectin (*SPARC*), runt-related transcription factor 2 (*RUNX2*) through quantitative real-time PCR (qRT-PCR). In brief, a commercial RNA extraction kit (TRIzol, Invitrogen, Carlsbad, CA, United States) was utilized to extract and collect total RNA from OB cells in the various studied groups. The extracted RNA was reverse transcribed to cDNA using an RNA-to-cDNA kit (Thermo Fisher, Waltham, MA, United States). Then, a SYBR™ Green Master Mix Kit (Thermo Fisher, Waltham, MA, United States) was used for performing qRT-PCR on a CFX96 real-time PCR detection system (BioRad, Hercules, CA, United States). Primer sequences used in this assay are shown in Table 1. *GAPDH* was regarded as a housekeeping gene.

**TABLE 1:** Primer sequences used for qRT-PCR.

Gene	Forward primer sequences	Reverse primer sequences
*RUNX2*	GAG​AGT​AGG​TGT​CCC​GCC​T	GAA​GTC​AGA​GGT​GGC​AGT​GT
*BGLAP*	GCA​GCC​TTC​GTG​TCC​AAG​CA	GCC​TCC​TGG​AAG​CCG​ATG​TGA​T
*SPP1*	ACC​GAT​CCG​ACG​AGT​CTC​ATC​AC	ACC​TCA​GTC​CAT​AGA​CCA​CAC​TAT​CC
*SPARC*	ACC​TGG​ACT​ACA​TCG​GAC​CTT​GC	GCT​TCT​CAT​TCT​CGT​GGA​TCT​CCT​TCA
*Col 1A1*	GAC​ATC​CCA​CCA​GTC​ACC​TG	CAC​CCT​TAG​CAC​CAA​CAG​CA
*ALPL*	AGC​CTT​CCT​GAA​AGA​GGA​TTG​G	GCC​AGT​ACT​TGG​GGT​CTT​TCT
*GAPDH*	GTG​AAG​GTC​GGA​GTG​AAC​GGA​TT	ACC​ATG​TAG​TGG​AGG​TCA​ATG​AAG​G

### 2.10 Statistical analysis

Experimental data are shown as the mean ± standard deviation (SD). Statistical analysis was carried out using SPSS 22.0 software (Armonk, NY, United States) and ORIGIN 9.0 software (Northampton, MA, United States). Student’s t-test and two-way ANOVA are applied to compare the significance among two groups or multiple groups. *p* < 0.05 was considered as the minimal level of significance, *p* < 0.01 was regarded as the medium level of significance, and *p* < 0.001 was indicated as the high level of significance.

## 3 Results

### 3.1 OB-EV_Dex_ characterization and cellular internalization

**FIGURE 1:**
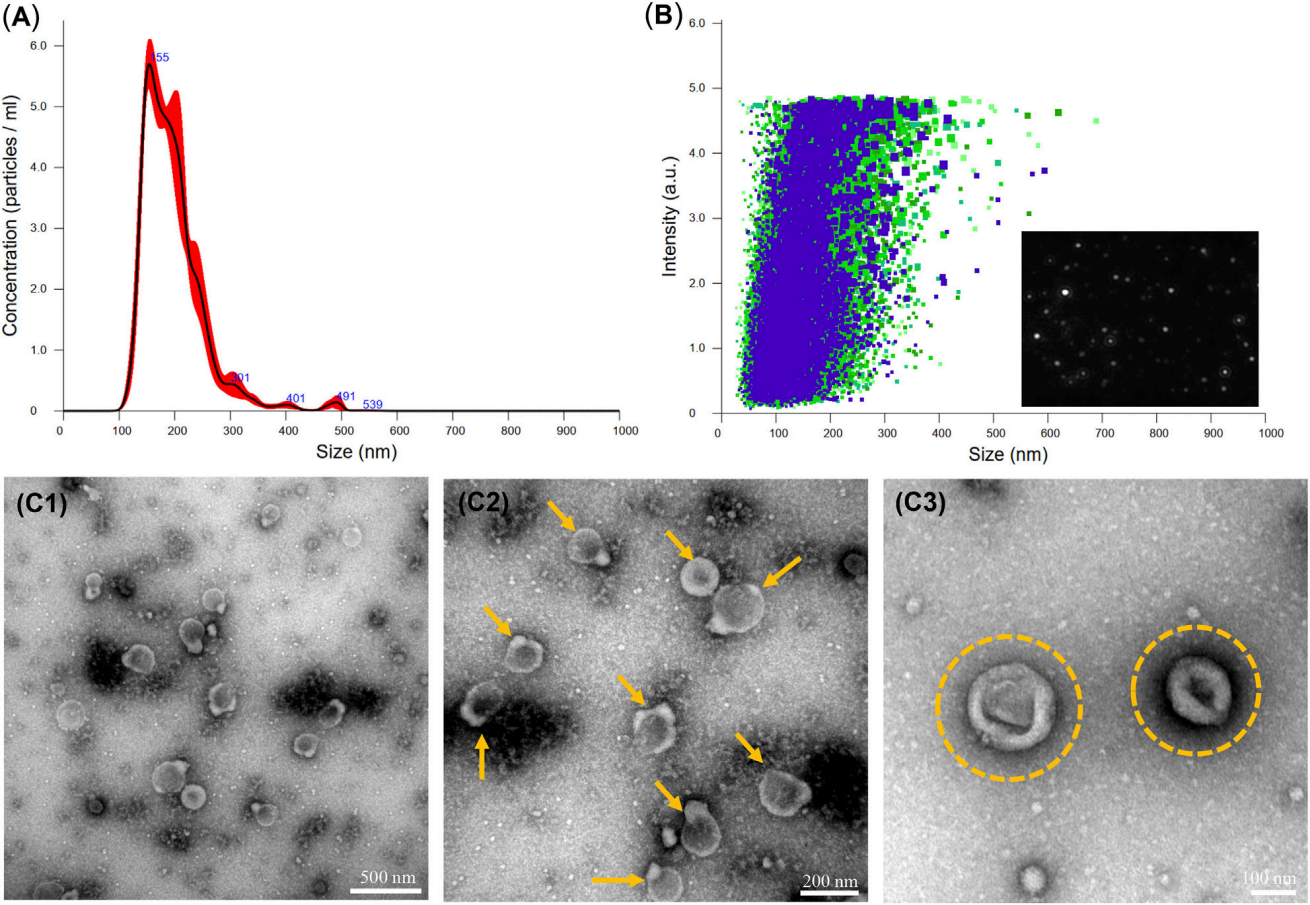
**(A, B)** Nanoparticle tracking analysis (NTA) of the obtained OB-EV_Dex_ revealing their size distribution, particle concentration, and intensity. **(C1-C3)** Transmission electron microscopy (TEM) images of isolated OB-EV_Dex_ at low and high magnification. Yellow arrows indicate the presence of OB-EV_Dex_; yellow circles indicate OB-EV_Dex_ showing a typical double-membrane nanostructure with spheroid shape.

The EV_Dex_ isolated from dexamethasone-stimulated OB cells were characterized for concentration and size distribution by NTA and TEM analysis. Figures 1A, B clearly verified the presence of a heterogeneous population of OB-EV_Dex_, mainly ranging from 100 to 500 nm, as revealed by NTA analysis. Of note, one primary population was identified, whose average diameter was roughly 178 ± 21 nm. After standardized OB-EV_Dex_ preparation, OB-EV_Dex_ concentrations were established to be around 6 × 10^9^ particles/mL in each fraction. The identity and sizes of OB-EV_Dex_ were further confirmed through TEM examination (Figures 1C1–1C3). This revealed the diameters of spherical vesicles to be 150–200 nm, which is in accordance with the NTA results. OB-EV_Dex_ exhibited a uniform distribution and a typical double-membrane nanostructure with spheroid shape, as also revealed by TEM analysis (Figure 1C3). These findings confirmed the successful isolation of OB-EV_Dex_.

**FIGURE 2:**
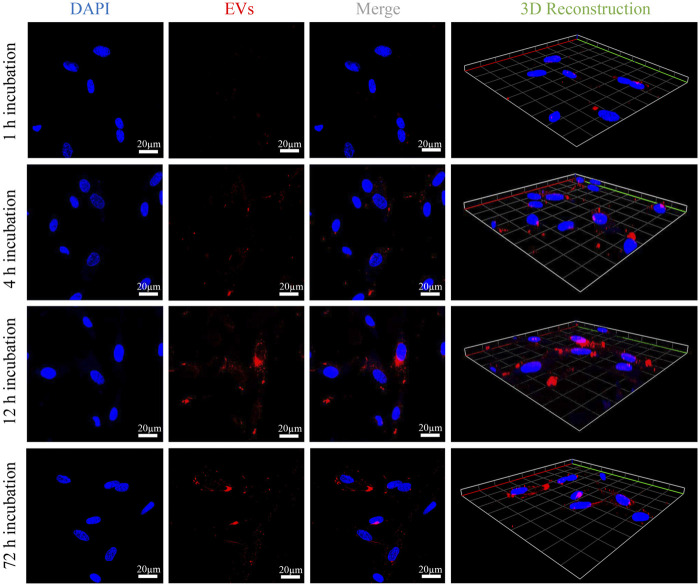
Confocal microscopy observations of cellular uptakes of fluorescently labeled OB-EV_Dex_ by osteoblasts after 1, 4, 12, and 72 h incubation. 3D reconstruction images of z-stack recorded by confocal microscopy. Fluorescently labeled OB-EV_Dex_ were detected as red fluorescence; cell nuclei were detected as blue fluorescence. Scale bar: 20 µm.

To justify that OB-EV_Dex_ could interact with OB cells, the cellular uptake of fluorescently labelled EV_Dex_ by OB cells was assessed by confocal laser scanning microscopy (CLSM). Figure 2 shows an apparent increment of the red fluorescence signal with prolonged incubation time (from 1 to 12 h). In fact, the red fluorescence signal from wheat germ agglutinin (WGA)-labeled OB-EV_Dex_ confirmed the presence of the EV_Dex_, that were assembled in the cell cytoplasm, validating that a large amount of labeled EV_Dex_ were taken up by OB cells. It was noteworthy that the increase in red fluorescence inside OB cells exhibited a time-dependent pattern. Of note, WGA-labeled red signals were the most intense when incubation time reached 12 h in comparison to other times. The 3D reconstruction images visually indicated the distribution of labeled EVs inside OB cells with increasing time. The majority of OB-EV_Dex_ were located in the cytoplasm and around the cellular nucleus. Notably, after 72 h post-incubation, there is a decrease in the intensity of red signals, attributable to the fact that these OB-EV_Dex_ were metabolized by the host cells.

### 3.2 Cytotoxicity assessment, cell attachment and proliferation of osteoblasts upon OB-EV_Dex_ stimulation

**FIGURE 3:**
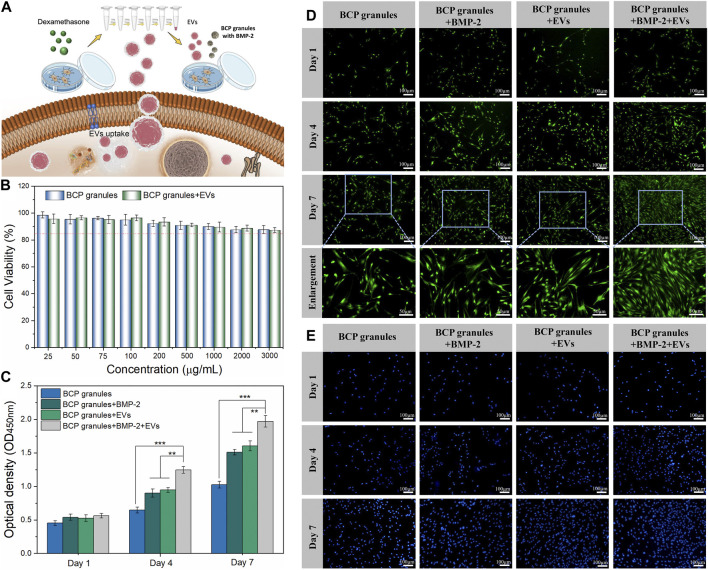
**(A)** Schematic illustration of key isolation steps of OB-EV_Dex_, internalization mechanism, and how OB-EV_Dex_ influence the osteogenic differentiation by transferring a complex cargo. **(B)** Cell viability of OB cells incubated with varying concentrations of bone granules suspension ranging from 25 to 3,000 μg/mL in the presence or absence of OB-EV_Dex_ (n = 3). Cell proliferation of OB cells on the BCP granules under various treatments measured by CCK-8 assay **(C)**, Calcein AM staining **(D)**, and DAPI staining **(E)** after 1, 4, and 7 days of culture (n = 3). Scale bar: 100 µm.

A schematic diagram of key isolation procedures, the internalization mechanism of OB-EV_Dex_, and how OB-EV_Dex_ influences osteogenic differentiation by transferring a complex cargo is sketched in Figure 3A. Prior to the cell experiments, the potential cytotoxicity of bone graft granules in the presence or absence of OB-EV_Dex_ was examined *via* the standard Cell Counting Kit-8 (CCK-8) assay. As shown in Figure 3B, the cell viability of osteoblasts after treatment of BCP granules suspension did not show an obvious decline in a wide concentration range from 25 to 3,000 μg/mL. Noticeably, the cell viability was constantly higher than 85% even at the highest concentration of 3,000 μg/mL with or without OB-EV_Dex_. These findings imply no present cytotoxicity of the bone BCP granules used nor of the OB-EV_Dex_, which is beneficial for applications with regard to osteoblast-dependent osteogenesis.

To prove cell attachment and proliferation on the bone BCP granules with or without OB-EV_Dex_, CCK-8 assay, DAPI staining, and Calcein AM staining were adopted to evaluate proliferative effects under various treatments after 1, 4, and 7 days of culture. As displayed in Figure 3D, the cell density (green fluorescence) significantly increased in various treatment groups with prolonged culturing time, especially on days 4 and 7 after seeding. The cell number and proliferation rate in the BMP-2+OB-EV_Dex_ group were significantly higher relative to other groups on the seventh day. Nevertheless, no apparent difference in cell number was observed between the BMP-2 group and the OB-EV_Dex_ group. Comparatively, the control group displayed the worst proliferation at all time-points. Besides, a similar uptrend of cell number in various treatment groups was detectable by DAPI staining assay (Figure 3E). The proliferative effects after various interventions were quantitatively examined by CCK-8 assay at designated time points (Figure 3C). At 4- and 7- days post-cultivation, optical density (OD value) of the BMP-2+OB-EV_Dex_ group was determined to be significantly higher than those of the BMP-2 and OB-EV_Dex_ groups (*p* < 0.01). Conversely, the control group had the lowest OD values at predetermined time intervals.

### 3.3 Influence of OB-EV_Dex_ on the viability, growth, and cell morphology

**FIGURE 4:**
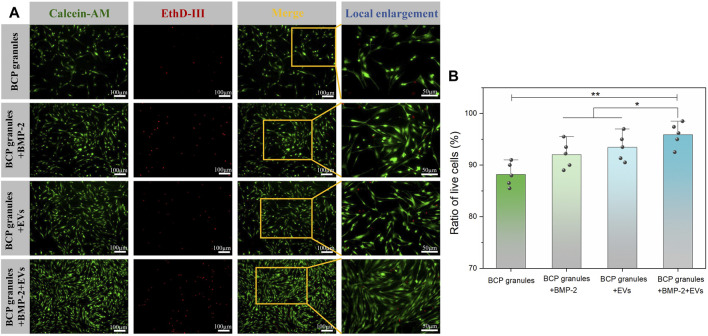
**(A)** Viability of OB cells under various interventions was evaluated by Calcein AM/EthD-III co-staining assay after 7 days of culture. Green fluorescence stands for live cells, and red fluorescence stands for dead cells. **(B)** Corresponding ratio of live cells versus dead cells, which was determined by ImageJ software at low magnification (×10) from five different random regions (n = 5).

The viability of OB cells under various interventions was evaluated by Calcein AM/EthD-III co-staining assay after 7 days of culture. Figure 4A showed that numerous live cells (green) were detected while a few dead cells (red) were scattered on these granules in the various treatment groups on the seventh day. The density of live cells was highest and pronouncedly amplified in the BMP-2+OB-EV_Dex_ group when compared to other treatment groups. Furthermore, the live cell and dead cell status were emphasized in local enlargement images. To directly determine cell viabilities under various interventions, the ratio of live cells to dead cells from five different randomly selected regions was calculated by the ImageJ software (Figure 4B). As expected, cell viability in the BMP-2+OB-EV_Dex_ group was highest, reaching 96.2%, whereas OB-EV_Dex_ and BMP-2 groups had lower viabilities, about 94.1%, and 92.8%, respectively.

**FIGURE 5:**
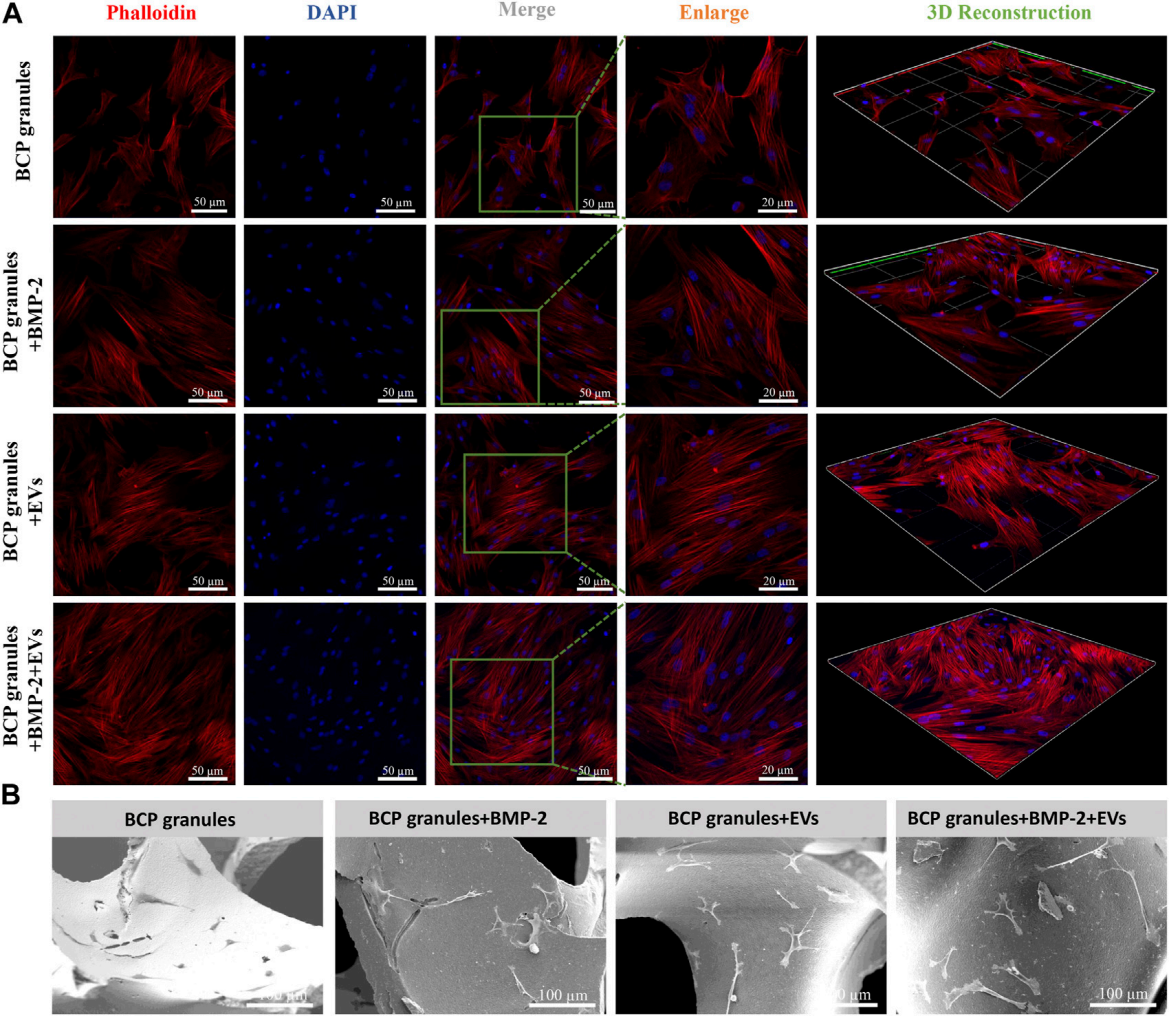
**(A)** Cell morphological observations of OB cells growth on bone BCP granules under various treatments after 7 days of cultivation *via* CLSM. OB cells were fluorescently labeled by cytoskeletal staining with Rhodamine Phalloidin and DAPI, which indicate F-actin (red) and cell nuclei (blue). Scale bar: 50 or 20 µm. **(B)** Representative SEM images of OB cells on bone BCP granules under various treatments after 7 days of cultivation. Scale bar: 100 µm.

Morphological traits of OB cells on bone BCP granular materials after 7 days of cultivation were examined by CLSM and SEM analysis. OB cells were fluorescently labeled by cytoskeletal staining with Rhodamine Phalloidin and DAPI, which indicated F-actin and cell nuclei (Figure 5A). Particularly, OB cells in the OB-EV_Dex_ group majorly presented a typical spindle-like morphology, with some filamentous pseudopodia; however, OB cells in the control group did not display such a typical morphology and apparently fewer cytoskeletons were found. Notably, an organized cytoskeleton network with a multitude of well-spreading OB cells was only detected in the BMP-2+OB-EV_Dex_ group. Meanwhile, these OB cells treated with the dual stimuli of BMP-2 and OB-EV_Dex_ displayed a relatively ordered distribution of the cytoskeleton layer along BCP granules.

SEM observations shown in Figure 5B indicated that OB cells in the BMP-2+OB-EV_Dex_ group exhibited a well-spreading morphology in response to the dual-stimuli. Moreover, OB cells developed an even stretched and elongated spindle-like shape with multiple prominent filopodia to tightly get hold on granules substrate surfaces. Conversely, OB cells in the granular materials group exhibited a polygonal morphology. No distinguishable difference in the cell morphologies could be identified between the BMP-2+OB-EV_Dex_ group and the OB-EV_Dex_ group.

### 3.4 Validation of osteogenic potential of OB-EV_Dex_ during osteogenic differentiation

**FIGURE 6:**
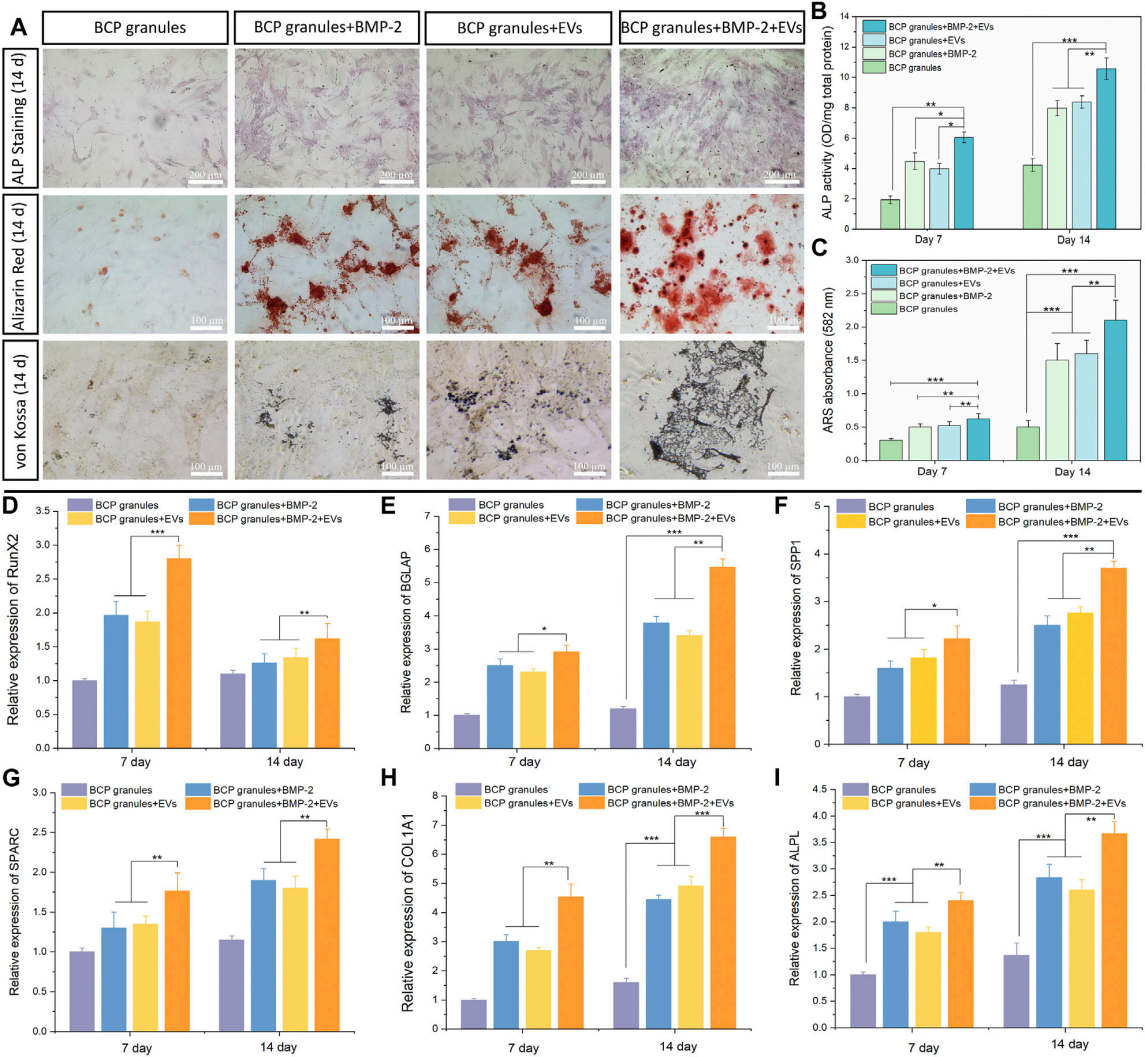
Osteogenic potential validation. **(A)** Biomineralization analysis of osteoblasts for different treatment groups measured by Alkaline Phosphatase staining, Alizarin Red staining and von Kossa staining on day 14. Scale bar: 100 or 200 µm. **(B)** Quantitative ALP activity and **(C)** colorimetric quantitative of Alizarin Red staining (ARS) after 7 or 14 days of culture (n = 3). Relative mRNA expression of selected osteogenic markers of **(D)** *RunX2*, **(E)** *BGLAP*, **(F)** *SPP1*, **(G)** *SPARC*, **(H)** *COL 1A1*, and **(I)** *ALPL* in osteoblasts after various treatments for 7 or 14 days, measured by quantitative RT-PCR (n = 3). *p < 0.05, **p < 0.01, and ***p < 0.001.

ALP staining and ALP activity were used to qualitatively and quantitatively analyze the osteogenic capacity of OB cells after various treatments after 7 and 14 days of cultivation. As illustrated in Figure 6A, the OB-EV_Dex_ and BMP-2 groups both produced a certain area of lavender-colored cobalt oxide precipitation on the 14th day. This confirms that EV_Dex_ or BMP-2 stimuli had a certain osteoinductive ability. Notably, the whole field of OB cells in the BMP-2+EV_Dex_ group was totally covered with lavender colored precipitation, suggesting that ALP levels were substantially elevated by the combined applications of OB-EV_Dex_ and BMP-2. Furthermore, ALP activities in all treatment groups progressively increased over the course of time (from 7 to 14 days). Besides, on the 14th day, ALP activities in the BMP-2+OB-EV_Dex_ group were significantly higher than those in the OB-EV_Dex_ and BMP-2 group (*p* < 0.05) by nearly 1.5 times (Figure 6B), while ALP activities in the OB-EV_Dex_ group was comparable to that in the BMP-2 group on either seventh or 14th day.

The mineralized nodules (calcium deposition) were detected by Alizarin Red and von Kossa staining after culturing for 2 weeks Figure 6A showed that small amounts of mineralized nodules were sparsely scattered in the OB-EV_Dex_ and BMP-2 groups, indicating the certain osteogenic potentials of OB-EV_Dex_ and/or BMP-2. In comparison, a substantial increase in cell-mediated calcium depositions (red or black precipitates) in the BMP-2+OB-EV_Dex_ group was observed on the 14th day. At that time, calcium deposition speckles appeared larger, reddish-brown, and tended to agglomerate on a large scale. In addition, according to the semiquantitative results of the Alizarin Red staining, we found that the BMP-2+OB-EV_Dex_ group produces much denser mineralized nodules and a higher degree of mineralization level on the 14th day than those in the OB-EV_Dex_ and BMP-2 group, as evidenced by absorbance at 582 nm (Figure 6C).

### 3.5 OB-EV_Dex_ significantly upregulate osteogenic marker gene expressions

The expression levels of major osteogenic marker genes of *RUNX2*, *BGALP*, *SPP1*, *SPARC*, *Col 1A1*, and *ALPL* in OB cells under the various studied interventions were measured on days 7 and 14 by performing qRT-PCR (Figures 6D–I). It was noted that *RUNX2* expression levels in all treatment groups were significantly higher on day 7 relative to day 14. In addition to *RUNX2*, significantly upregulated expressions of selected genes of *Col 1A1*, *ALPL*, *BGALP*, *SPP1*, and *SPARC* were noted over the course of time. Notably, the BMP-2+OB-EV_Dex_ treatment resulted in a remarkably elevated expression of *Col 1A1* (Figure 6H) relative to the BMP-2 and OB-EV_Dex_ groups (*p* < 0.01). *BGLAP* and *SPP1*, two typical markers of late-stage biomineralization, exhibited an extremely similar time-dependent uptrend with *Col 1A1* expressions, especially for the BMP-2+OB-EV_Dex_ group (Figures 6E, F). Besides, it was evident that on either day 7 or 14, mRNA expression levels of *ALPL* in the OB-EV_Dex_ group were comparable to those of the BMP-2 group. These findings mentioned above were in accordance with biomineralization experiments.

## 4 Discussion

Substantial research has uncovered that MSCs-derived EVs exhibited great promise in regenerative medicine since they are able to induce lineage-specific stem cell differentiation and accelerate new bone tissue formation and regeneration (Alqurashi et al., 2021; Hade et al., 2021). Despite numerous studies reporting “the specific roles of MSC-derived EVs”, however, it is surprising that, to date, only a very limited number of investigations deal with the role of osteoblast-derived EVs in bone biology (Cappariello et al., 2018; Man et al., 2022).

In the present study, we provided insights into some of the potentials of EVs secreted from consecutive 14 days of dexamethasone-stimulated osteoblasts (OB-EV_Dex_) for osteogenesis. With regard to OB-EV_Dex_ endocytosis, it was observed that a sufficient amount of fluorescently labeled OB-EV_Dex_ were taken up by porcine osteoblasts in a time-dependent pattern and mainly distributed in the perinuclear region. This is in line with the concept that effective cellular uptakes of extracellular vesicles are of vital significance for performing biological effects (Tkach and Théry, 2016).

Considering that the capacity for proliferation, attachment, and migration of transplanted cells is of most significance in boosting tissue regeneration (Monaco et al., 2011; Ma et al., 2019), we, therefore, evaluated the proliferative effects of OB-EV_Dex_ on osteoblast cells. Our findings have demonstrated that OB-EV_Dex_ significantly enhanced OB surface attachment and proliferation when compared to untreated cells. Interestingly, OB-EV_Dex_ treatment resulted in a comparable pro-proliferative effect with that found in the BMP-2 group. The obtained results of the control and BMP-2 group are in line with findings known from the literature (Luppen et al., 2003) and therefore indicated that the found results for the OB-EV_Dex_ treatment group are not related to any artifact. The increased cell number and proliferation rate in the BMP-2+OB-EV_Dex_ group compared to the BMP-2 or OB-EV_Dex_ group demonstrate that the cell proliferation effect was obviously strengthened due to the synergistic amplifying effect of OB-EV_Dex_ and BMP-2. This contradicts some of the known effects of dexamethasone in regard to the suppression of osteoblast proliferation but, on the other hand, shows possible counteracting effects of OB-EV_Dex_ restoring not only late-phase but also early-phase osteogenic effects. In addition, cell viability is a vital metric for the evaluation of cellular behaviors (Zhang et al., 2019). We speculate that the high cell viability (96.2%) found in the BMP-2+OB-EV_Dex_ group might be attributed to the synergistic amplifying proliferative effect supporting the cell with proteins driving the early and late onset of ossification. Furthermore, osteoblast-derived EVs were recently shown to regulate cell-to-cell communication such as by stimulating and facilitating the recruitment of endogenous cells, which greatly benefits bone tissue restoration (Man et al., 2021).

Cellular morphological traits influenced by osteogenic microenvironments are regarded as a vital indicator in assessing the cell-material/microenvironment interactions (Zhu et al., 2021). Our study found that OB cells in the OB-EV_Dex_ group developed an even stretched and elongated spindle-like morphology with some filamentous pseudopodia to get hold on BCP granules substrate surfaces more tightly. In addition, OB cells in the control group only exhibited a polygonal morphology. Accumulating evidence indicates that the filamentous pseudopodium is highly involved in cell adhesion, stretching, spreading, and proliferation (Yom-Tov et al., 2014; Yan et al., 2019; Chen et al., 2021). Interestingly, a mass of adhered osteoblasts with a well-organized cytoskeleton network was only found in the BMP-2+OB-EV_Dex_ group. This might be due to a combinational stimulating effect from OB-EV_Dex_ and BMP-2, which might induce more filopodia formation supporting cell adhesions to granules matrix surfaces and simultaneously making the OB cells produce an extremely dense cytoskeleton layer throughout the cells themselves.

Nevertheless, biological mineralization is an extremely complex, multiple-stage biological process (Matta et al., 2019). In our study, the results of intensity and number of mineralized nodules (calcium deposition) indicate that BMP-2+OB-EV_Dex_ treatment exhibited a greater pro-osteogenic potency than solely OB-EV_Dex_ or BMP-2 treatment. This suggests a crucial role of dual-stimuli from OB-EV_Dex_ and BMP-2 in strengthening osteoblastic differentiation of OB cells by elevating calcium deposition levels, likely due to the significant enrichment of pro-mineralization proteins associated with OB-EV_Dex_, including, e.g., annexins as reported by Davies et al. (2019).

Our findings provided strong evidence that the combinations of OB-EV_Dex_ and BMP-2 were considerably superior at enhancing ALP activities when compared to OB-EV_Dex_ or BMP-2 alone. This suggests that the combination of OB-EV_Dex_ and BMP-2 presents increased pro-osteogenic effects, especially at the early or middle phase of osteogenic differentiation and mineralization (Vimalraj, 2020). Moreover, our study discovered that *RUNX2* expression is positively influenced in the OB-EV_Dex_-treated groups. This could be explained by the fact that *RUNX2* is a crucial transcription factor that activates the early osteogenesis differentiation phase (Bruderer et al., 2014), which is an essential step for the initiation of bone regeneration. The upregulation of *RUNX2* expression might be one of the closest explanations for the counteracting effect of OB-EV_Dex_ towards the suppressive effect of dexamethasone. In addition, it was observed that the combination of EV_Dex_ and BMP-2 engenders a maximum activating effect to elevate *Col 1A1* matrix protein expression levels, thereby facilitating the maturation of osteogenic mineralization (Yan et al., 2019; Man et al., 2021). In addition to *RUNX2* and *Col 1A1*, the expression of other selected marker genes of *ALPL*, *BGLAP*, *SPP1*, and *SPARC* all exhibited a significant upregulation over time, especially for the BMP-2+OB-EV_Dex_ group. In contradiction to that, Uenaka et al. (2022) found that differentiated osteoblasts release a subset of small EVs in order to inhibit bone formation and enhance osteoclastogenesis. These findings might not be applicable to what we found here, due to the fact that they focused on native OB-EVs and a different subset of EVs (diameter of 200–400 nm).

Taken together, our findings reported here revealed that OB-EV_Dex_ significantly amplify pro-osteogenic processes while promoting the osteoblastic differentiation by upregulating the expressions of crucial osteogenesis-related marker genes, as well as mineralization nodules and ALP activity. Besides, it is noteworthy that the most apparent pro-osteogenic differentiation capacity in upregulating gene expressions was evoked by OB-EV_Dex_ together with BMP-2. On the basis of above evidence, we reasoned that irrespective of any clinical challenges, the prospective use of dexamethasone-induced OB-EV_Dex_ could function as a novel osteogenic accelerator for treating, or at least alleviating the symptoms of bone defects in future clinical practice. It may be possible that OB-EV_Dex_ can be a promising candidate that circumvent the limitations associated with traditional cell-based therapies, such as limited cell sources, immunogenicity, low survival of transplanted cells. Altogether, this study provides a solid foundation for advancing OB-EV_Dex_ towards the clinical translation.

Although our study has demonstrated the pronounced potential of OB-EV_Dex_ to act as a new modified biological tool for improving osteogenesis, it still presents a few restrictions. First, we did not identify the most exact components inside OB-EV_DEX_ responsible for improving the *in vitro* osteogenesis, and therefore, further research is required for the exploration the underlying mechanism. Second, we did not assess the influence of OB-EV_Dex_ on bone restoration and regeneration *in vivo*, which may provide better direct evidence about the therapeutic potential of OB-EV_Dex._ Nevertheless, the findings reported in this study are crucial for EVs-related osteogenesis in the frame of bone tissue engineering.

## 5 Conclusion

In our study, dexamethasone-stimulated osteoblast-derived EVs (OB-EV_Dex_) could markedly promote osteoblastic differentiation by positively upregulating crucial osteogenic genes, but also significantly augment capacities for *in vitro* proliferation, attachment, and viability of osteoblasts. Of note, the pro-osteogenic effects mediated by OB-EV_Dex_ were comparable to those of individual BMP-2 treatment. In addition, the combinational applications of OB-EV_Dex_ and BMP-2 were more advantageous in stimulating proliferation, differentiation, and biomineralization, when compared to OB-EV_Dex_ or BMP-2 treatment alone, resulting in notable increases in calcium nodules during osteogenesis. Taken together, our findings elucidate the potential of dexamethasone-induced OB-EV_Dex_ as a prospective therapeutic option for enhancing osteogenesis.

## Data Availability

The original contributions presented in the study are included in the article/supplementary material, further inquiries can be directed to the corresponding authors.
